# Decellularized extracellular matrix-based disease models for drug screening

**DOI:** 10.1016/j.mtbio.2024.101280

**Published:** 2024-09-27

**Authors:** Zhoujiang Chen, Ji Wang, Ranjith Kumar Kankala, Mingli Jiang, Lianlin Long, Wei Li, Liang Zou, Aizheng Chen, Ya Liu

**Affiliations:** a Institute for Advanced Study, Chengdu University, Chengdu, 610106, Sichuan, PR China; b Affiliated Hospital & Clinical Medical College of Chengdu University, Chengdu University, Chengdu, 610106, Sichuan, PR China; c Institute of Biomaterials and Tissue Engineering, Huaqiao University, Xiamen, 361021, Fujian, PR China; d School of Pharmacy, Zunyi Medical University, Zunyi, 563099, Guizhou, PR China

**Keywords:** Drug screening, Extracellular matrix, Cell models, Decellularization, Personalized medicine

## Abstract

*In vitro* drug screening endeavors to replicate cellular states closely resembling those encountered *in vivo*, thereby maximizing the fidelity of drug effects and responses within the body. Decellularized extracellular matrix (dECM)-based materials offer a more authentic milieu for crafting disease models, faithfully emulating the extracellular components and structural complexities encountered by cells *in vivo*. This review discusses recent advancements in leveraging dECM-based materials as biomaterials for crafting cell models tailored for drug screening. Initially, we delineate the biological functionalities of diverse ECM components, shedding light on their potential influences on disease model construction. Further, we elucidate the decellularization techniques and methodologies for fabricating cell models utilizing dECM substrates. Then, the article delves into the research strides made in employing dECM-based models for drug screening across a spectrum of ailments, including tumors, as well as heart, liver, lung, and bone diseases. Finally, the review summarizes the bottlenecks, hurdles, and promising research trajectories associated with the dECM materials for drug screening, alongside their prospective applications in personalized medicine. Together, by encapsulating the contemporary research landscape surrounding dECM materials in cell model construction and drug screening, this review underscores the vast potential of dECM materials in drug assessment and personalized therapy.

## Graphical abstract

**Figure:**
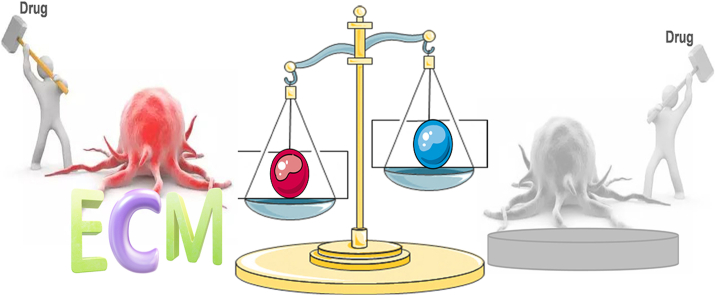

## 1 Introduction

The development of novel pharmaceuticals is an inherently high-risk endeavor that demands substantial financial investment and time commitment. Within the phases of drug efficacy evaluation and safety assessment, cellular models play a pivotal role in shaping research outcomes [1]. Considerably, cellular models provide invaluable insights into prospective drug candidates, encompassing their biological functionality and the possibility of adverse reactions. Thus, the meticulous selection and proficient utilization of appropriate cellular models are of paramount significance in determining the effectiveness and safety profiles of drugs towards innovative drug development.

In the conventional drug evaluation process, monolayered cell cultures in two-dimensional (2D) models offer simplicity and facilitate high-throughput screening. Although uncomplicated and support efficient drug assessment at scale, these 2D models suffer from limitations, including the challenges of replicating the complex *in vivo* cellular microenvironments as well as the alterations in cellular morphology, metabolic pathways, and gene expression, among others [2]. In 2D cultures, the tissue culture plates or glass surfaces that come into contact with cells are much stiffer than human soft tissues, potentially altering cellular behavior and phenotypes [3]. Hence, three-dimensional (3D) cell culture has emerged as a more robust cellular model for drug evaluation. In 3D cell culture, cells are cultivated within a matrix or scaffold composed of various materials, such as hydrogels, biodegradable polymers, or natural extracellular matrix (ECM) components [[4], [5], [6]]. These matrices provide structural support to the cells, enabling them to grow and interact in a manner that closely mimics their *in vivo* behavior. 3D cell culture has become an indispensable tool in biological research and drug development. Compared to 2D cultures, 3D cultures generally exhibit lower drug sensitivity in most cases during the evaluation of anti-cancer drugs [7]. These models enable scientists to examine cells in a physiologically relevant context, enhancing their understanding of cellular behavior and allowing for a more precise assessment of the interactions between candidate drugs and cells.

ECM is a 3D structure surrounding cells, composed of a complex molecular network, including proteins, polysaccharides, and other biomolecules [8]. ECM serves several crucial biological functions. Firstly, it provides physical support to cells, allowing them to maintain specific shapes and arrangements, thereby contributing to the overall structural integrity of tissues. Secondly, proteins within the ECM can bind to cell surface receptors, promoting cell-ECM adhesion, which is associated with cell migration. Lastly, specific molecules within the ECM can act as signaling molecules, influencing cell growth, differentiation, and function, thereby regulating cell behavior [9]. Consequently, ECM-based biomaterials hold significant value in studying the interactions between cells and tissues and in recapitulating the physiological state and biological signals of cells in their *in vivo* environment. These attributes play pivotal roles in constructing ECM-based cell models for drug assessment and evaluation. In our previous study, recellularization of liver decellularized extracellular matrix (dECM) with HepG2 cells significantly promoted the epithelial-mesenchymal transition (EMT) process more effectively compared to 2D culture, enhancing the drug resistance of HepG2 cells [10].

**Fig. 1:**
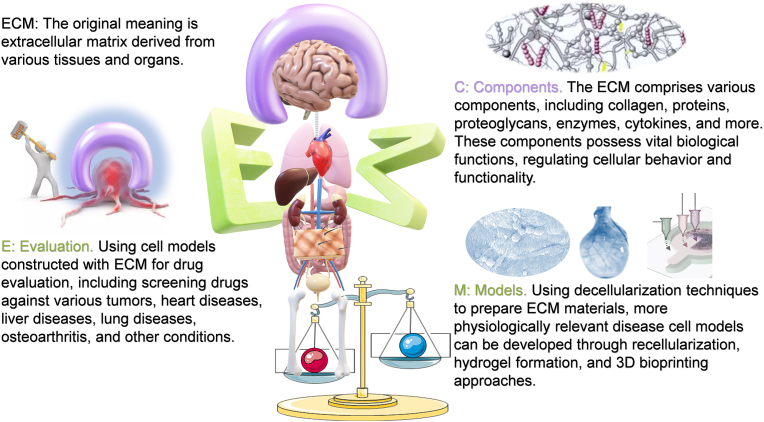
Schematic illustration of the research on the preparation of cell models using ECM of various organ tissues in drug screening.

This review aims to explore the application of ECM-based biomaterials in the field of drug assessment (Fig. 1). Firstly, we introduce the characteristics of ECM, including its components and their functional roles. Subsequently, we analyze the techniques for ECM preparation, discussing the advantages and disadvantages of each method and their applications. Then, the article focuses on the pivotal discussion of ECM materials in the construction of various tissue and organ models, as well as their relevance in drug assessment. Finally, we summarize the article with the prospects and challenges of ECM-based materials in the field of drug assessment.

## 2 The biological functionality of extracellular matrix components

The mammalian ECM is a multifaceted structure composed of various components, including proteins, polysaccharides, enzymes, and ECM-binding growth factors, among other bioactive molecules [11]. Biological tissues exhibit a tightly regulated structural organization, resulting in the formation of distinctive 3D morphologies for ECM components [12]. ECM plays a crucial role in biological functions, involving interactions not only among ECM components but also between these components and cells. This section aims to provide a comprehensive overview of the biological functionalities of key ECM components and their association with tissues or diseases, potentially guiding the selection of ECM materials in the construction of disease models and drug screening processes.

### 2.1 Collagens and proteins

Collagens, as structural proteins pervasive within tissues, confer formidable mechanical resilience, endowing tissues with the capacity to withstand tension, compression, and cyclic stressors [13]. They are pivotal for creating and maintaining essential spatial structures within the cellular growth environment. Collagens are widely distributed across various anatomical sites, including tendons, skeletal and cartilage tissues, the dermis, and the interstitial ECM enveloping organs. In the majority of tissues and organs, collagen constitutes over 95 % of the dry weight of the ECM. To date, researchers have identified 28 distinct types of collagens, each showcasing unique functionalities [14]. For instance, type I collagen exhibits remarkable tensile strength due to the abundance of ligaments and tendons, enhancing the mechanical load-bearing capacity of tissues [15]. In contrast, other collagen types are less prevalent, yet each type fulfills specific roles within different tissue microenvironments. Type II collagen predominates in cartilage, while type III collagen forms a delicate network of reticular fibers widely dispersed in elastic tissues, such as skin, blood vessels, and loose connective tissues within organs. Type IV collagen adopts a 2D mesh-like configuration, serving as a key constituent of basement membranes and scaffolds. Meanwhile, type VI collagen, characterized by its relatively small size, acts as a bridging unit between glycosaminoglycans (GAGs) and larger structural proteins, such as type I collagen, imparting a gel-like viscosity to the ECM. Finally, type VII collagen is situated in the basement membrane of the epidermis, where it functions as an anchoring fiber, contributing to protective mechanisms [15].

Typically, collagen serves as a foundational scaffold for cellular support, with its mechanical properties potentially influencing cellular behaviors. Notably, the proliferation of cancer cells can be modulated by the stiffness of the ECM, a process tightly regulated by an array of signaling cascades [16,17]. In addition, the intricate interplay between collagen and cellular signaling pathways intricately governs cell fate across diverse tissue contexts and pathological states. Previous reports suggested that collagen could engage with a repertoire of at least five distinct cell receptors, including integrins, discoidin domain receptors (DDR), glycoprotein VI, osteoclast-associated receptor (OSCAR), leukocyte-associated immunoglobulin-like receptor-1 (LAIR-1), and uPARAP/Endo180. These dynamic interactions could significantly contribute to modulating diverse cellular functions [18].

Integrins, heterodimeric receptors abundantly adorning the cell surface, wield paramount influence in orchestrating cell signaling, migration, viability, and differentiation attributes. Collagen receptors, including integrins α1β1, α2β1, α10β1, and α11β1, exhibit specific affinities for collagen, thereby modulating discrete cellular signaling pathways [19]. Notably, integrins α1β1, α2β1, and α11β1 emerge as pivotal mediators in the reconstitution of collagen, particularly accentuated in processes vital for wound healing [20]. The engagement of collagen with the integrin α1β1 receptor intricately regulates cellular proliferation, matrix metalloproteinase expression, and collagen biosynthesis [21]. Meanwhile, integrin α2β1 stands out as a principal collagen-binding integrin prevalent in bone, skin, and visceral organs, displaying a predilection for fiber isoforms of collagen I, II, III, V, and XI [22]. Concomitantly, integrin α10β1 assumes prominence as the primary receptor for type II collagen, predominantly found in cartilage tissues [23]. The intricate interplay between collagen and integrins is of pivotal significance across diverse physiological and pathological contexts, spanning cardiac hypertrophy, oncogenesis, EMT, and tumor dissemination [18]. Hence, the regulatory prompt of collagen in cellular signaling warrants meticulous consideration in the design of cellular models.

Within the mammalian ECM, alongside collagen proteins, a lot of other proteins coexist, each wielding distinct physiological functionalities (as depicted in Table 1). For instance, elastin assumes a fibrous form within tissues, including the skin, lungs, bladder, heart, and aorta. The elastin furnishes structural support to withstand repetitive stretching, thereby aiding in shape restoration [24]. However, elastin, under pathological circumstances, may undergo enzymatic degradation, yielding elastin peptide fragments, impacting the onset and progression of vascular diseases and cancer [25]. To this end, fibronectin engages in multifaceted interactions with various proteins, carbohydrates, and cell surface receptors, such as integrins and syndecan, thereby exerting pivotal roles in modulating cell adhesion, proliferation, migration, differentiation, and wound healing processes [26]. Notably, the heightened expression of fibronectin in the matrix surrounding tumor vasculature potentially fosters tumor advancement [27]. Laminin, another ubiquitous constituent of the ECM, populates the basal lamina, assuming a critical role in facilitating cell adhesion and migration during early embryonic development. Furthermore, laminin mediates cell-cell interactions through diverse receptor-mediated mechanisms [28]. Its upregulation in injured epithelial tissue fosters epithelial regeneration and augments vascular growth [29]. Notably, mutations in laminin chains underlie various human congenital disorders, including congenital muscular dystrophy and epidermolysis bullosa [30]. Moreover, investigations have elucidated interactions between laminin and other basement membrane components as well as cell surface receptors, with such interactions facilitated by PI-3K and RAC1 activation demonstrated to potentiate the invasion of squamous cell carcinoma cells [31].

**Table 1:** Brief description of functions of major proteins in the ECM.

Collagen and proteins	Functional description
Collagen	With 28 distinct types, it provides mechanical support, rigidity, and affinity for cell ligands, acting as a linking molecule and interacting with other ECM components.
Elastin	Provides support for the restoration of shape under repetitive stretching conditions. Under pathological conditions, elastin can be degraded by enzymes, which affects the occurrence and development of vascular diseases and cancer.
Fibronectin	Regulating cell adhesion, growth, migration, and differentiation, as well as wound healing.
Laminin	Plays a crucial role in cell adhesion and migration during early embryonic development. Upregulated expression is observed in injured epithelium, promoting epithelial regeneration and vascular growth. Also associated with the invasion of squamous cell carcinoma cells.
Thrombospondin	Play functional roles in angiogenesis, bone formation, wound healing, and myocardial remodeling, among others.
SPARC	Induced and involved in processes such as wound healing, sites of angiogenesis, and tumor formation.
Osteopontin	Upregulated in sites of inflammation and tissue remodeling, where it plays a role in cell adhesion and migration. Also involved in the pathogenesis of diseases such as atherosclerosis, glomerulonephritis, and cancer.
Tenascin-C	Highly expressed under pathological conditions such as chronic inflammation, heart failure, atherosclerosis, and cancer, inducing cell migration, proliferation, and modulation of signaling pathways.

In the intricate milieu of tissues, matricellular proteins emerge as a distinctive class of non-structural extracellular modulators orchestrating cellular functions (as delineated in Table 1). This heterogeneous group encompasses thrombospondins, tenascins, secreted protein acidic and rich in cysteine (SPARC), osteopontin, cartilage oligomeric matrix protein, periostin, fibulin, pigment epithelium-derived factor, among others, acting as conduits for intercellular communication and mediating interactions between cells and the ECM. For instance, thrombospondins are implicated in diverse physiological processes spanning angiogenesis, bone formation, wound healing, myocardial remodeling, and more [32]. SPARC, abundantly expressed during mammalian development and tissue differentiation, exhibits diminished expression in mature organs yet is induced in contexts such as wound healing, sites of angiogenesis, and tumorigenesis, potentially serving as an inhibitor of adipogenesis [33]. Osteopontin, upregulated in sites of inflammation and tissue remodeling, exerts influence over cell adhesion and migration *via* integrin interactions, contributing to the pathogenesis of conditions such as atherosclerosis, glomerulonephritis, and cancer [34]. Similarly, Tenascin-C, prominently expressed in pathological settings like chronic inflammation, heart failure, atherosclerosis, and cancer, modulates cellular behaviors, including migration, proliferation, and signaling cascades through mechanisms entailing pro-inflammatory cytokines and oncogenic signaling molecules [35].

### 2.2 Glycosaminoglycans and proteoglycans

GAGs represent a complex array of linear polysaccharides comprised of disaccharide units containing uronic acid and hexosamine. This diverse category encompasses heparin, chondroitin sulfate, heparan sulfate, keratan sulfate, dermatan sulfate, hyaluronan, and others. The composition and distribution of GAGs within tissues undergo dynamic alterations across various processes, including aging, acute injuries, thrombotic disorders, and age-related tissue degeneration [36]. Notably, distinct GAG components harbor specific biological functionalities. Heparin and heparan sulfate, for instance, find application as pharmaceutical agents for conditions characterized by heightened blood clotting. Endothelial cells typically secrete sulfated heparin, which binds antithrombin III to the vascular wall, thus impeding platelet adhesion and coagulation factor activity [37]. Furthermore, heparins engage in interactions with vascular endothelium-derived growth factor (VEGF), fibroblast growth factor (FGF), and epidermal growth factor (EGF), thereby modulating the functions of these associated factors [[38], [39], [40]]. Chondroitin sulfates, categorized based on sulfate group positioning, such as chondroitin sulfate A and C prevalent in joints, contribute to lubrication owing to their abundant sulfate groups [41].

Additionally, chondroitin sulfate can activate ECM-degrading enzymes like matrix metalloproteinases [42]. In tumor contexts, the binding of chondroitin sulfate to P-selectin enhances cancer cell adhesion to vascular endothelial cells, fostering tumor progression and metastasis [43]. Keratan sulfate exhibits increased expression in ocular, osseous, and cerebral tissues. Notably, with advancing age, keratan sulfate chain length and sulfation degree tend to grow within the ECM [44]. Dermatan sulfate assumes roles in collagen assembly regulation, mesenchymal stem cell and neural stem cell maturation induction, and bone-forming cell differentiation modulation [[45], [46], [47]]. Hyaluronic acid (HA), a pivotal ECM constituent constituting over 50 % of skin composition, is indispensable for hydration, lubrication, ECM structural integrity maintenance, and plasma protein distribution control [48]. Moreover, HA exerts crucial biological functions, including dendritic cell maturation stimulation, cell proliferation, and migration regulation, with implications in diseases such as colitis, osteoarthritis, and cancer progression [49,50].

Proteoglycans (PGs) represent a class of macromolecules characterized by a protein core adorned with the aforementioned GAG modifications, ubiquitous within both cellular and ECM compartments. Beyond furnishing structural support in tissues, PGs assume multifaceted roles as structural elements or ligands for growth factors, cytokines, chemokines, and beyond, exerting regulatory control over crucial processes spanning embryonic development, inflammatory responses, and intercellular communication [51]. Notably, a conspicuous presence of aggrecan is evident within the ECM of cartilaginous tissues like articular cartilage and trachea. Functioning as an aggregating PG tethered to HA, aggrecan bolsters the formation of a resilient and pliable structure within the collagen protein framework of cartilage [52]. Similarly, perlecan, found within the basement membrane and adjacent cells, binds to heparan sulfate, interacting with an array of components, including laminin, type IV collagen, and fibronectin, thereby contributing to tissue structure maintenance [53]. In addition, perlecan serves as a reservoir for certain growth factors and, *via* interactions with VEGF, orchestrates processes, such as angiogenesis promotion [54]. Small leucine-rich proteoglycans (SLRPs) assume pivotal roles in tissue organization, imparting orderliness critical for specific tissue functionalities, notably evident in structures like tendons and the cornea [55].

### 2.3 Others

The ECM harbors a plethora of pivotal bioactive constituents, including enzymes, cytokines, and growth factors, pivotal for orchestrating tissue development, repair, regeneration, and homeostasis processes. Continuous ECM remodeling, involving protein degradation and deposition, is imperative for maintaining tissue integrity. Among these remodeling agents, matrix metalloproteinases (MMPs) emerge as a crucial enzyme class, pivotal for ECM homeostasis maintenance and autocrine-paracrine signaling regulation. Notably, MMP-1 has been implicated in inducing osteogenic differentiation of mesenchymal stem cells, thus fostering bone tissue regeneration [56]. Moreover, MMPs are involved in the pathophysiology of diverse ailments, including cancer, cardiovascular diseases, osteoarthritis, and emphysema. For instance, MMPs play crucial roles in tumor invasion, metastasis facilitation, and tumor angiogenesis [57]. In addition, mediators, such as growth factors and cytokines, wield profound influence over cellular behavior, engaging in close interplay with the ECM. Notably, cytokines and ECM reciprocally modulate each other's expression and synthesis. Certain cytokines and growth factors exhibit direct binding affinity to specific ECM components, sequestering within the matrix for subsequent release [58].

The ECM boasts a vast repertoire of proteins, numbering in the hundreds, encompassing collagen proteins, fibronectin, proteoglycans, and osteopontin, among others. These proteins serve as stalwart pillars, endowing cells and tissues with structural support and resilience. Complementing the proteinaceous constituents, the ECM hosts polysaccharide molecules, such as hyaluronic acid and chondroitin sulfate, which are pivotal for water absorption and maintaining the gel-like ECM structure. Furthermore, the ECM houses an assortment of bioactive molecules, including growth factors and extracellular matrix-binding proteins, wielding regulatory sway over cellular behavior and functionality. Hence, the components of the ECM are involved in and regulate cellular activities, providing significant biological advantages over synthetic polymers.

## 3 Preparation of cell models based on ECM materials

### 3.1 Decellularization techniques for obtaining ECM materials

In native tissues, cells intricately interweave with the ECM. In this context, the cell components, including cell membranes, organelles, and nuclear constituents containing cell antigenic epitopes, serve as pivotal triggers for host responses, such as inflammation and immune rejection [59]. Hence, the elimination of cellular elements stands as a pivotal phase in the preparation of ECM-derived biomaterials. The decellularization process of tissues and organs is meticulously crafted to expunge cellular constituents to the utmost degree while safeguarding the quintessential components, biological activity, and structural integrity of the native ECM (as depicted in Table 2). The evolution of decellularization technology in the 1970s has found application across medical domains encompassing reconstructive surgery, organ transplantation, and regenerative medicine [60]. Since its evolution, the dECM has emerged as a compelling class of biomaterials with promising prospects.

**Table 2:** Summary of commonly used decellularization methods and their advantages and disadvantages.

Strategy		Mechanism	Pros and Cons	Ref.
Physical	Freeze-thaw	The cellular formation of ice crystals disrupts the cell membrane	The degradation of ECM components is typically mild and minimal, and incomplete decellularization often requires subsequent washing and enzymatic digestion.	[61]
	Mechanical pressing	Promotes the separation of cells from the underlying basement membrane		[8]
	Ultrasound	Disrupt the structure of cell membranes		[62]
	Supercritical fluid	Dissolves and removes lipid components of the cell membrane	Specific equipment is required. While it preserves ECM components relatively well, subsequent washing is still necessary.	[63]
Chemical	Acid/base treatment	Disrupt cell membranes, dissolving and removing cellular and nuclear components.	Damages the ECM structure, affecting GAG and collagen components. Potential antimicrobial effects.	[64,65]
	Detergents	Dissolves membrane components, leading to the separation of substances such as DNA and proteins.	Common decellularization methods are relatively simple but may damage or alter the ECM structure, reducing ECM components.	[[66], [67], [68], [69], [70]]
	Hypotonic/hypertonic solutions	Induces cell lysis and dehydration through osmotic effects	Difficult to remove DNA remnants.	[71]
Enzymatic	Nuclease	Degrades RNA and DNA.	After physical or chemical treatment, nucleases are commonly used to remove residual nucleic acid components, reducing immunogenicity.	[8]

Several methods for eliminating cellular components from tissues encompass a spectrum of approaches spanning physical, chemical, and enzymatic techniques, as comprehensively documented in prior literature [8]. Firstly, the physical-based methodologies for cellular component removal imply various techniques, such as repeated freeze-thaw cycles, mechanical pressing, ultrasound, and supercritical fluid technology. Freeze-thaw cycles induce the formation of intracellular ice crystals, culminating in cell membrane disruption [61]. Ultrasound primarily disrupts cell membrane structures, while mechanical pressing aids in the separation of cells from the underlying basement membrane, facilitating decellularization [62]. Furthermore, the supercritical fluid technology stands out as an advanced decellularization technique, boasting environmental friendliness owing to carbon dioxide's status as a green solvent, obviating the need for hazardous chemicals. Within a supercritical carbon dioxide milieu, lipid constituents of cell membranes readily dissolve, thereby achieving efficient decellularization. Previous reports suggested that dECM materials prepared utilizing supercritical fluid technology exhibited superior preservation of ECM components compared to conventional chemical methods [63]. However, reliance solely on physical methods might not entirely eradicate residual cell membranes and cellular remnants, necessitating subsequent washing and enzymatic treatment steps for thorough purification.

Secondly, chemical methods stand as efficient strategies for decellularization, encompassing acid-base treatments, detergent washes, and solutions with high or low osmolarity. These chemical methods facilitate biomolecule hydrolysis and cell membrane disruption. Acid-base treatments that are effective in biomolecule hydrolysis efficiently dissolve and remove cellular and nuclear components, often boasting antimicrobial properties. For instance, peroxyacetic acid serves as a standard disinfectant with decellularization capabilities [64]. Conversely, several harsh chemical agents, including sodium sulfide and sodium hydroxide, can disrupt collagen crosslinks, leading to a significant reduction in the mechanical strength of dECM [65]. Detergents achieve decellularization by dissolving cell membranes and facilitating DNA dissociation from proteins. These detergents include ionic agents (sodium dodecyl sulfate, SDS) and non-ionic agents, such as Triton X-100 and zwitterionic detergents. Triton X-100 accelerates tissue delipidation compared to ionic detergents. At the same time, SDS proves more effective in removing nuclear residues and cytoplasmic proteins, particularly in dense tissues or organs like the liver or kidney, than non-ionic agents [66,67]. Several zwitterionic detergents like CHAPS and SB-10 find extensive utility in preparing decellularized tissues from blood vessels, nerves, and lungs [[68], [69], [70]]. Alternating hypotonic and hypertonic solutions disrupt DNA-protein interactions, facilitating cell dissolution. This method is commonly employed for thinner tissues, such as the cornea [71]. For larger and thicker structures or whole organs, perfusion serves as an effective decellularization technique [72]. Sequential perfusion of surfactants (e.g., SDS) or cleansing solutions into blood vessels effectively removes cellular components while largely preserving vital organ structures such as vascular channels.

Finally, the enzymatic treatment offers a viable option for dECM preparation, achieved by disrupting cell-to-cell and cell-to-ECM interactions using nucleases, proteases, collagenases, lipases, and other enzymes, leading to specific removal of cellular remnants and efficient cell eradication. However, enzymatic methods typically incur significant damage to ECM components, thereby compromising ECM mechanical strength [8]. Furthermore, residual enzymatic byproducts may incite inflammation, induce cell apoptosis, and provoke immune rejection during subsequent dECM material preparation [73]. It should be noted that the dECM preparation relying solely on the enzymatic treatment often results in insufficient comprehensive decellularization, necessitating combined approaches with physical or chemical methods. For instance, subsequent to decellularization with surfactants, supplementary processing with DNA enzymes becomes imperative to address residual nucleic acid molecules, notorious for triggering immune responses [10].

### 3.2 Common approaches for constructing cell models based on ECM materials

**Fig. 2:**
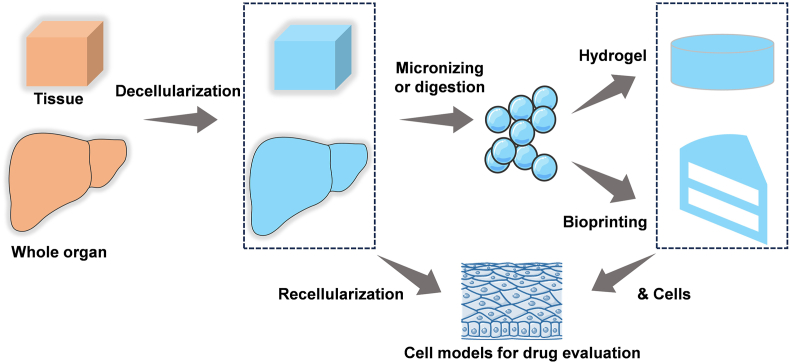
Approaches for constructing cell models based on dECM. This includes directly recellularizing tissues and whole organs after decellularization to construct cell models, digesting or microfabricating decellularized tissues to prepare hydrogels, and using 3D bioprinting to build disease models.

The inherent constituents within the ECM serve as foundational support for reconstructing native cellular states when constructing *in vitro* cell models. The direct utilization of ECM recellularization or the integration of ECM biomaterials as scaffolds has emerged as a potent method for constructing *in vitro* cell models (Fig. 2). This approach finds diverse applications in regenerative medicine and disease modeling, encompassing organs such as the liver, ovary, blood vessels, and pancreas, among others [10,[74], [75], [76]]. Furthermore, the dECM derived from various tissues and organs has been harnessed to produce bioinks for application in bioprinting to fabricate *in vitro* tissue models [77]. This process entails the physical micronization of ECM tissue or enzymatic digestion of ECM using pepsin, a commonly employed technique. Pepsin selectively cleaves peptide bonds at the ends of collagen triple helical structures, thereby disassembling collagen fibril aggregates. The resultant digested ECM solution can gel upon pH and temperature adjustment [78]. Several composite scaffolds have been fabricated by incorporating synthetic polymers into the ECM to overcome deficiencies, such as poor mechanical strength of ECM. These innovative strategies find broad utility in tissue engineering and regenerative medicine endeavors [79].

## 4 Application of ECM in drug screening

### 4.1 Screening of antitumor drugs

Solid tumors exhibit complex organ-like structures comprising tumor cells, vascular networks, ECM, stromal elements, and immune cells, among others [80]. In the process of screening anticancer drugs, the conditions of *in vitro* cell culture affect drug sensitivity, thereby influencing the identification of clinically effective candidate drugs [81]. Typically, tumor models are created by directly culturing tumor cells and constructing them into 2D or 3D structures. During the construction of 3D tumor models, various materials, such as Matrigel or fibrous scaffolds, can be used to promote cell aggregation and the formation of 3D morphology. Within the tumor microenvironment, the ECM plays a pivotal role, profoundly impacting critical processes, such as tumorigenesis, progression, metastasis, and drug resistance, thereby exerting a considerable influence on therapeutic outcomes [82]. Consequently, a growing body of research is focusing on unraveling the intricate role of the ECM in the realm of cancer therapy. The advent of tumor culture systems leveraging decellularized ECM-based materials harbors significant promise for advancing drug development endeavors.

#### 4.1.1 Liver-derived dECM

Hepatocellular carcinoma (HCC) stands as a prevalent malignant digestive disorder, with its global incidence steadily increasing. Sun et al. have innovated a hybrid gel bead incorporating both liver ECM and alginate for *in vitro* 3D cell culture [83]. The integration of ECM markedly augmented the expression of urokinase plasminogen activator system proteins and matrix metalloproteinase activity in human hepatocellular carcinoma HCCLM3 cells, thereby establishing an *in vitro* research model conducive to investigating liver cancer metastasis and screening anti-metastasis drugs. Chemotherapy remains pivotal in HCC management, driving extensive research into chemotherapeutic agent development. In an instance, Bao colleagues cultured HepG2 cells on a substrate derived from the decellularized porcine liver matrix, observing enhanced cell proliferation, increased albumin secretion, and urea synthesis compared to cells cultured on Matrigel and collagen type I substrates [84]. Furthermore, 3D cellular aggregates formed on the decellularized liver matrix micropattern array chips facilitated quantitative assessment of paclitaxel, doxorubicin, and disulfiram toxicity *via* fluorescence quantitative analysis, emphasizing the suitability of this cellular microsphere approach for rapid and convenient drug susceptibility testing [84]. In another instance, Woo and colleagues established a liver cancer model by perfusing HepG2 hepatocellular carcinoma cells through the portal vein post-decellularization of rat liver tissue [85]. Compared to 2D and spheroid culture models, cells cultivated on this model displayed heightened expression of E-cadherin, matrix metalloproteinase 9, and multidrug resistance protein 2 while exhibiting diminished impact on albumin secretion and alpha-fetoprotein expression in response to methotrexate. This model closely mimics the *in vivo* cellular state, facilitating the evaluation and prediction of novel anticancer drug efficacy. Recently, our group generated a liver cancer model by recellularizing decellularized liver tissue with HepG2 cells [10]. Compared to 2D culture, this cell model exhibited increased levels of albumin secretion and urea synthesis and promoted the EMT process in HepG2 cells. Notably, doxorubicin showed some degree of resistance in this cell model, while triptolide and honokiol exhibited less apparent resistance, potentially attributed to their ability to reverse the EMT process.

**Fig. 3:**
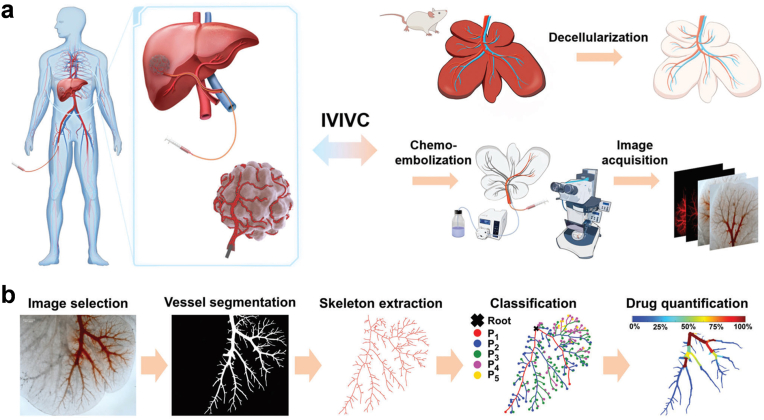
Construction of decellularized rat liver-based drug release model to establish in vitro–in vivo correlations with in-human results in TACE. (a) Drug-loaded embolic agents are injected into one main branch of portal vein vasculature to mimic the superselective treatment of TACE. (b) Spatiotemporal drug release quantitative calculation of intravascular drug concentration. Reproduced with permission from Ref. [86], Copyright © 2023 Wiley-VCH GmbH.

Transarterial chemoembolization (TACE) emerges as a promising modality for treating unresectable HCC. This therapeutic approach entails the administration of drug-eluting embolic agents to occlude tumor-feeding arteries, thereby facilitating direct delivery of chemotherapy into the tumor. Nonetheless, significant debate persists regarding optimal treatment parameters, necessitating further elucidation of drug release dynamics within the tumor milieu. Recently, Guo et al. demonstrated the perfusion of rat liver with detergent solutions to decellularize it, laying the groundwork for the development of an organ-structured drug release model [86]. In their investigation, they replicated the TACE treatment paradigm by injecting drug-loaded embolic agents into a major branch of the portal venous system within this model (Fig. 3). Comparing two drug-loaded embolic agents—namely, the liquid embolic emulsion of EO-DOX and the microparticle-based embolic agent of drug-eluting beads—they scrutinized their release profiles within the model. The analysis of release kinetics yielded valuable insights and recommendations for enhancing the efficacy of these embolic agents.

#### 4.1.2 Breast-derived dECM

Breast cancer has emerged as one of the most prevalent malignant tumors among women worldwide, with survival rates presenting a less-than-optimistic outlook. Establishing suitable *in vitro* breast cancer models holds pivotal significance in advancing our comprehension of the biological intricacies of breast cancer and expediting drug development efforts. Typically, decellularized breast tissue and breast adipose tissue serve as scaffolds or recellularized constructs, effectively emulating breast cancer cell characteristics and furnishing a valuable platform for drug assessment. Engel et al. harnessed bioprinting technology to craft a breast tumor model comprising a hybrid matrix incorporating porcine breast ECM, GelMA, and alginate [87]. Structurally, this model comprises an inner core housing MCF-7 cells and an outer layer composed of stromal cells, thus faithfully simulating tumor tissue. To enhance the mimicry of the ECM milieu within breast tumors, type I collagen was integrated into the bioink, resulting in augmented tumor cell proliferation and diminished sensitivity to doxorubicin [87]. In addition, the adipose tissue ECM offers a biologically relevant microenvironment for breast cancer cells. In a case, An and colleagues leveraged human adipose tissue-derived ECM as a scaffold to establish a 3D cell culture system for tumor cells, encompassing MCF-7, BT474, and SKBR3 breast cancer cells [88]. The resultant tumor models exhibited growth characteristics and cellular organization more akin to xenografts. Notably, different cells cultured on the scaffold manifested distinct responses to drugs. Compared to 2D culture, breast cancer (MCF-7 and BT474) models demonstrated increased resistance to doxorubicin, whereas SKBR3 and BT474 breast cancer cells exhibited augmented sensitivity to lapatinib, attributing to elevated phosphorylation levels of epidermal growth factor receptor and protein kinase B.

#### 4.1.3 Lung-derived dECM

Lung cancer is broadly classified into two classes such as non-small cell lung cancer (NSCLC) and small cell lung cancer (SCLC), in which NSCLC represents approximately 85 % of cases, and SCLC constitutes the remaining 15 % [89]. Alongside surgical interventions, chemotherapy is one of the most prevalent adjunctive treatment modalities for lung cancer, particularly in cases of metastatic SCLC. The ECM components within lung tissue exert significant influence on regulating the phenotype and function of cells, including fibroblasts. Notably, researchers uncovered that inhalation of decellularized lung ECM could confer protection against hypoxia-induced lung injury [90]. Delving into ECM-mediated signaling pathways holds paramount importance in comprehending lung tumor metastasis and facilitating drug assessment in lung cancer. Consequently, researchers endeavored to develop *in vitro* lung ECM mimetic models by formulating hydrogels incorporating diverse lung ECM-related peptides [91]. Leveraging decellularized lung ECM as a biomaterial to replicate the lung tissue environment for anticancer drug evaluation could promise to deepen our insights into the tissue microenvironment's impact on drug efficacy.

**Fig. 4:**
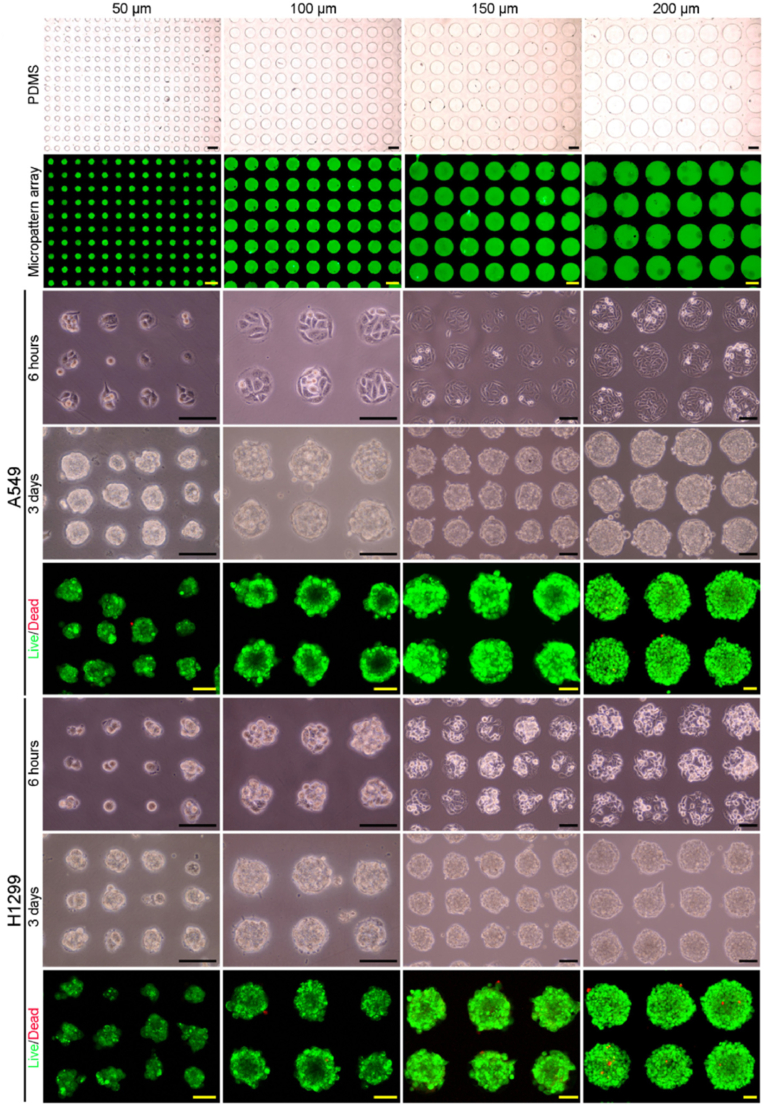
Fabrication of different-sized porcine lung dECM-based hydrogel-coated micropattern arrays and the formation of cancer cell spheroids. Reproduced with permission from Ref. [93], Copyright © 2022 Elsevier.

In the pursuit of constructing *in vitro* models of lung tumors, the integration of lung dECM not only facilitates angiogenesis but also faithfully replicates the natural microenvironment of tumor tissue. In a case, Jung and colleagues innovated a vascularized lung tumor chip utilizing porcine lung dECM hydrogels, incorporating A459 cells, human lung fibroblasts, and human umbilical vein endothelial cells [92]. The lung cancer simulation platform exhibited notable distinctions in cytotoxicity responses to varying doxorubicin concentrations compared to 2D cell models. In a 2D culture, cells exhibited relatively higher levels of doxorubicin-induced p53 protein expression, which was devoid of dose-dependent characteristics compared to the dECM model. In contrast, the lung cancer tissue-like model showcased dose-dependent p53 expression levels in response to doxorubicin, affirming its improved sensitivity to the drug in this model. Precision in controlling the size of 3D cell microspheres holds paramount importance in the construction of drug screening models, ensuring reproducibility and accuracy in drug evaluation. In another case, Li et al. engineered four different-sized (ranging from 50 μm to 200 μm) porcine lung dECM-based hydrogel-coated micropattern arrays for 3D cellular models utilized in drug screening (Fig. 4) [93]. It was corroborated that lung dECM as the microarray model provided superior support for lung cancer cell adhesion, distribution, vitality, and proliferation in comparison to type I collagen and Matrigel. The 3D models, comprising A549 and H1299 cells, exhibited resistance to paclitaxel, doxorubicin, and cisplatin when juxtaposed with 2D cell models. However, these distinct cell sources demonstrated variations in cytotoxic responses to the drugs, with H1299 cells showcasing heightened sensitivity to doxorubicin and cisplatin but reduced sensitivity to paclitaxel.

#### 4.1.4 Brain-derived dECM

**Fig. 5:**
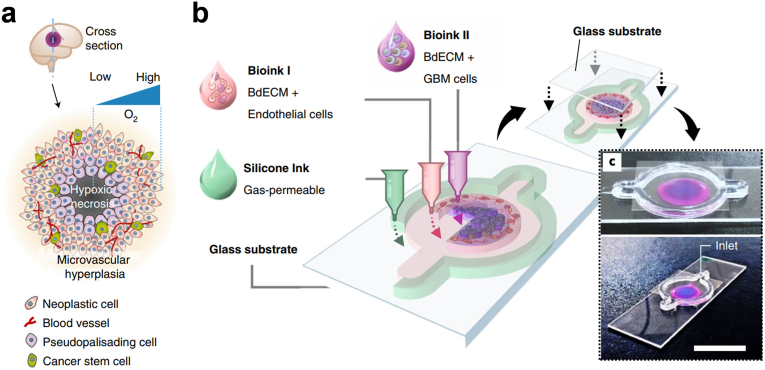
(a) Schematic illustration of the microenvironment of glioblastoma. (b) The process for printing the glioblastoma-on-a-chip model with a compartmentalized structure using various bioinks and other materials. Reproduced with permission from Ref. [95], Copyright © 2019 Springer Nature.

In neuro-oncology, glioblastoma multiforme (GBM) has emerged as the most prevalent and lethal brain malignancy, constituting approximately 50 % of all malignant primary brain tumors [94]. To scrutinize the pathological attributes and microenvironment of native glioblastomas *in vitro*, dECM derived from brain tissue serves as a valuable resource for constructing tumor models. In this context, Cho and colleagues harnessed patient-derived glioblastoma cells, vascular-associated cells, and porcine brain dECM as bioink for 3D printing, thereby establishing an *ex vivo* tumor-on-a-chip model [95]. This chip faithfully replicated the hypoxic microenvironment associated with the dense growth of glioblastomas, with cancer cell aggregates enveloping the surrounding microvasculature to stimulate a central hypoxic region, thereby mirroring the pathological features of glioblastomas (Fig. 5). The chip model replicated the drug resistance patterns similar to those clinically observed in patients following radiotherapy and temozolomide treatment while also exploring potential drug combinations effective against tumors. Considering the diverse molecular profiles and sensitivities exhibited by patient-derived cancer cells in various *in vitro* culture environments, the development of *ex vivo* models mirroring the pathological attributes of the primary tumor enabled the screening of promising candidate drugs. In another recent study, Li and coworkers introduced a novel approach employing decellularized mouse brain slices for glioblastoma cultivation, which more faithfully recapitulated the transcriptome of glioblastoma cells compared to traditional 2D culture methods [96]. This approach demonstrated the influence of brain ECM on glioblastomas, including its role in augmenting glioma cell glucose consumption and lactate production, enhancing resistance to temozolomide, and influencing glioma cell-mediated ECM degradation.

#### 4.1.5 Tumor-derived dECM

Each organ and tissue boasts unique ECM compositions, including tumors, tailored to fulfill tumor-specific roles and functions. Utilizing tumor-derived dECM as a scaffold material for *in vitro* cell models holds promise in faithfully replicating the tumor microenvironment. This approach meticulously mimics the *in vivo* state of tumor cells accurately, thereby enabling more precise assessments of drug efficacy.

Decellularized breast cancer tissue from patients conserves the native structure and constituents of tumor tissue, thus facilitating the replication of intricate tumor tissue microenvironments. Landberg and coworkers prepared patient-derived scaffolds from breast cancer specimens obtained from 15 patients and recellularized with various breast cancer cell lines, including MCF-7, T-47D, and MDA-MB-231 cells, thereby offering *in vivo*-like microenvironments for drug screening [97]. The experimental results revealed that MCF-7 cells cultured on these scaffolds displayed heightened resistance to 5-fluorouracil, doxorubicin, and paclitaxel compared to 2D culture systems. Notably, high doses of doxorubicin diminished cancer stem cell characteristics, while 5-fluorouracil intensified stem cell properties while reducing the proliferative phenotype. Conversely, T-47D and MDA-MB-231 cells exhibited growth patterns akin to MCF-7 cells while stimulated with drugs at IC50 concentrations in both culture systems. However, following 5-fluorouracil and doxorubicin treatment, MDA-MB-231 cells cultured in 2D systems displayed reduced total RNA levels compared to those on scaffolds. In addition, T-47D cells exhibited enhanced drug response and gene expression in 2D cultures relative to scaffold cultures, indicating that the microenvironment provided by the scaffold influences drug responses, with different cell lines impacting specific gene expression in chemotherapy. In another study, Dai and colleagues decellularized human breast cancer biopsy tissues extracted from multiple patients using a sodium dodecyl sulfate (SDS) solution, followed by recellularization with MCF-7 cells [98]. The designed model not only augmented cell proliferation and migration but also facilitated EMT in cells, along with exhibiting resistance to 5-fluorouracil compared to 2D cultures. The stiffness of tumor tissue varies from that of normal tissues and the surrounding tumor microenvironment, potentially influencing tumor progression [99]. Zhao and colleagues utilized transfection techniques to modulate lysyl oxidase expression levels in MDA-MB-231 cells, resulting in the generation of tumors with differing ECM stiffness when implanted in animals. Following decellularization and subsequent recellularization of the tumor ECM scaffold *ex vivo*, it was observed that scaffolds with higher stiffness displayed increased resistance to cisplatin and higher expression of drug-resistant genes [100].

Pancreatic cancer has emerged as one of the most lethal malignancies, often diagnosed at an advanced stage, thereby limiting the efficacy of therapeutic interventions [101]. The pancreatic cancer microenvironment prominently displays a desmoplastic response, fostering tumorigenesis and conferring resistance to radiotherapy and chemotherapy [102]. Leveraging dECM from pancreatic cancer as a scaffold, the development of an *in vitro* 3D model mirroring the tumor microenvironment offers promise for screening effective drugs against pancreatic cancer. In a recent study by Agostini et al., decellularization of pancreatic cancer tissues obtained from multiple patients through surgical resection at a hospital was performed to create *in vitro* 3D scaffolds for cell culture [103]. This scaffold faithfully recapitulated the ultrastructure of the pancreatic tumor microenvironment, with mass spectrometry analysis revealing compositional disparities compared to normal pancreatic tissue. Remarkably, this 3D tumor model exhibited resistance to 5-fluorouracil relative to 2D cells and validated the heightened cytotoxicity of FOLFIRINOX regimens for pancreatic cancer.

### 4.2 Screening of cardiac disease drugs

Heart disease presents a formidable health challenge, accounting for numerous heart failure patients and exhibiting resistance to conventional drug therapies [104]. Consequently, there is an urgent need to devise tailored medications and personalized strategies. Among such strategies, cardiac tissue engineering has garnered enormous interest from researchers as a pivotal frontier [105]. Typically, myocytes in the human heart constitute approximately 20%–30 % of the total cell population. In contrast, the remaining non-myocytes include endothelial cells, fibroblasts, and smooth muscle cells [106]. Immature cardiac cells not only beat spontaneously but also possess an inherent ability to form 3D functional syncytia, making the fabrication of cardiac tissues relatively simple. Currently, human cardiomyocytes derived from pluripotent stem cells have been widely utilized and combined with biomaterials, such as hydrogels and matrices to construct models. These models hold significant potential for drug screening and patient-specific disease modeling. Previous studies indicated that, during *in vitro* culture, ECM could enhance the structural organization of cardiomyocytes and promote the expression of cardiac genes and proteins in cardiac progenitor cells [107]. Thus, the ECM of the heart retains the native 3D architecture and intricate matrix components, offering inherent advantages for fabricating cardiac tissue and disease models [108].

**Fig. 6:**
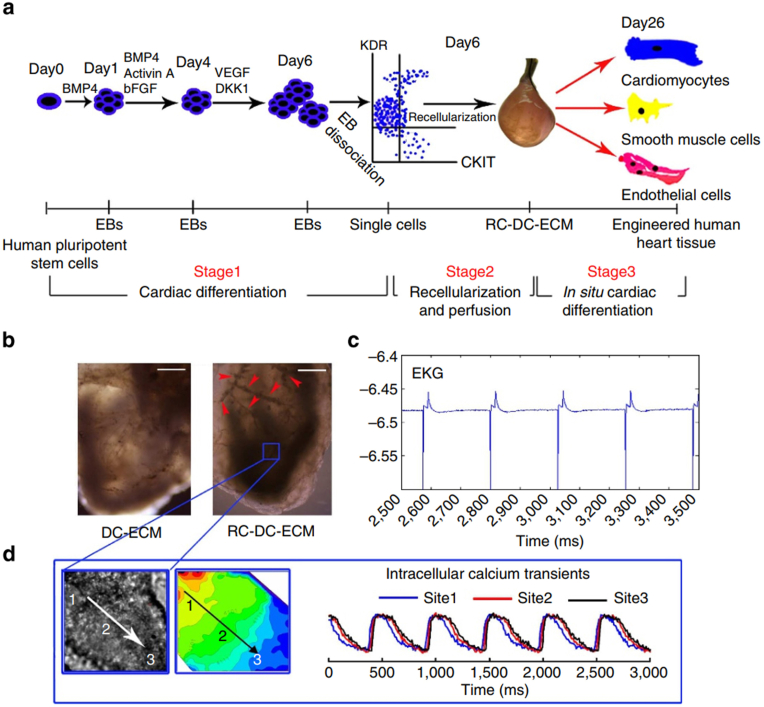
(a) The approach involves recellularizing decellularized mouse hearts using human iPSC-derived multipotential cardiovascular progenitors. (b) Identification of vessel-like structures, (c) Recording of electrocardiograms (EKG), and mapping of calcium transients (CaiT) in the recellularized tissue. Reproduced with permission from Ref. [109], Copyright © 2013 Springer Nature.

In a groundbreaking study, Yang and colleagues decellularized whole mouse hearts and subsequently repopulated them with human induced pluripotent stem cell (hiPS) -derived multipotential cardiovascular progenitors [109]. This approach validated the migration and differentiation capabilities of these progenitor cells into cardiomyocytes, smooth muscle cells, and endothelial cells, culminating in the construction of a functional heart model capable of spontaneous contraction and mechanical force generation (Fig. 6). Upon stimulation with isoproterenol, an escalation in heart contraction frequency was observed. In a study, isoproterenol was used as a selective β1-adrenergic agonist to investigate its stimulation of the chronotropic response in recellularized mouse hearts, confirming the success of personalized heart constructs. Furthermore, the cardiac model developed in this study was expected to emerge as a pioneering platform for elucidating arrhythmias and facilitating drug assessments in the future. Tung et al. reported the development of engineered heart slices by decellularizing porcine myocardium slices and seeding them with human-induced pluripotent stem cell-derived cardiomyocytes. The dECM slices guided cell alignment and enabled long-term culture, exhibiting positive inotropic responses to isoproterenol, anisotropic conduction of action potentials, and electrophysiological functions [110]. They also investigated the cardiac sensitivity to ion channel-modulating drugs, revealing differences compared to monolayer cell cultures, demonstrating the potential of this model for long-term electrophysiological and drug studies.

The biochemical milieu orchestrated by the ECM assumes a pivotal role in directing the differentiation of cardiac progenitor cells and facilitating cardiac reprogramming. Regarding the utilization of whole heart dECM and heart slices scaffolds, hydrogels, and bioinks incorporating cardiac ECM stand as essential modalities for modeling heart disease. Cho and coworkers ingeniously integrated heart-derived ECM into 3D hydrogels, effectuating a chemical reprogramming that fosters fibroblast-to-cardiomyocyte conversion [111]. On the one hand, decellularized heart ECM is likely to offer an ideal integrin-ECM interaction for cardiac lineage specification. On the other hand, in this cardiac micro-tissue, chemically induced cardiomyocytes exhibited enhanced electrophysiological characteristics and improved responsiveness to isoproterenol. Treatment with 100 nM and 1 μM isoproterenol increased the beats per minute by 135.7 % and 163.3 %, respectively. Thus, this strategy provided a robust platform for heart disease modeling and drug screening. In an instance, Gepstein and colleagues synergistically amalgamated ECM hydrogels enriched with chitosan alongside hiPSC-derived cardiomyocytes (hiPSC-CMs), culminating in the fabrication of engineered heart tissues characterized by anisotropic muscle organization and augmented cardiomyocyte maturity [112]. This model enabled the evaluation of drug effects on contraction rate, optical signal morphology, cellular arrhythmogenicity, and tissue conduction properties, encompassing a spectrum of drugs, such as isoproterenol, carbamylcholine, E−4031, ATX2, ouabin, lidocaine, carbenoxolone, and quinidine. For instance, treatment with 1 μM isoproterenol increased the force of contraction by 42 % and significantly shortened the contraction and relaxation times of engineered heart tissues. Lidocaine, on the other hand, markedly slowed conduction by approximately 38 %. Additionally, Kim and collaborators utilized a hydrogel scaffold composed of porcine myocardial extracellular matrix and reduced graphene oxide to culture human-induced pluripotent stem cells, thereby modulating the mechanical and electrical properties of the hydrogels [113]. This approach not only significantly upregulated the expression of genes associated with twitch forces and contractile function in hiPSC-derived cardiomyocytes but also improved electrophysiological functions, including calcium handling, action potential duration, and conduction velocity. Interestingly, cisapride, a hERG channel blocker known to cause arrhythmias showed no arrhythmogenic properties in preclinical drug screening but exhibited action potential prolongation and abnormal changes in beat intervals in this model, aligning with clinical responses. Leveraging this methodology to fabricate bioprinted cardiac tissues, they scrutinized the proarrhythmic properties induced by cisapride, thereby furnishing a versatile platform for delineating the cardiac adverse effects of drugs.

### 4.3 Screening of liver disease drugs

The liver stands as a pivotal organ within the human body, orchestrating vital functions such as protein synthesis, blood detoxification, and immune regulation. Liver-related ailments, encompassing hepatitis, liver failure, injury, and fibrosis, pose formidable threats to human well-being. To comprehensively evaluate drug efficacy, the construction of apt *in vitro* models of liver diseases proves indispensable. The liver contains two main types of cells: parenchymal cells (hepatocytes) and non-parenchymal cells (such as Kupffer cells and hepatic stellate cells). Primary hepatocytes are commonly used in short-term human *in vitro* liver models for evaluating drug-induced hepatotoxicity. Hepatic stellate cells are considered to play a crucial role in the process of fibrosis. Under normal conditions, these cells are in a quiescent state. However, upon exposure to stimuli, such as inflammation and liver injury, they become activated and transition into proliferative myofibroblast-like cells. Therefore, researchers typically constructed an *in vitro* liver fibrosis model by culturing hepatic stellate cells either alone or in co-culture with hepatocytes, followed by the induction of injury [114]. Liver fibrosis models were then employed for large-scale screening of pro-fibrotic or anti-fibrotic drugs. Additionally, exposing hepatocytes to a free fatty acid environment is also commonly used to construct non-alcoholic fatty liver disease models [115]. Leveraging liver ECM for the generation of liver microtissues and artificial liver constructs holds promise in simulating liver function and bolstering liver cell-specific functionality within *in vitro* models [116]. In a recent study by Mandal et al., a mixture of porcine liver ECM, silk fibroin, and gelatin was used as bioink to construct a human physiomimetic liver acinus model [117]. This model was designed to assess drug-induced liver injury that exhibited clinically relevant characteristics and functions. The ECM component played a crucial role in enhancing cell viability, colonization, and proliferation in the model. Employing decellularized liver ECM for the fabrication of 3D-printed liver structures induces stem cell differentiation, thereby augmenting the functionality of HepG2 cells [118]. Introducing primary human liver cells into decellularized mouse livers yields miniature human liver constructs, manifesting the expression of liver-specific mRNA [119]. Kang and colleagues fabricated a composite by incorporating gelatin into porcine liver dECM material to enhance printability, thereby fabricating liver lobule-like structures imbued with endothelial cell-like architecture and primary hepatocytes [120]. Baharvand and coworkers devised liver-derived ECM hydrogels from decellularized sheep liver tissue, demonstrating that hepatocarcinoma cells cultivated in these hydrogels exhibited an epithelial phenotype with marked upregulation of liver-specific transcripts compared to traditional 2D cultures and 3D collagen gel organoids [121].

**Fig. 7:**
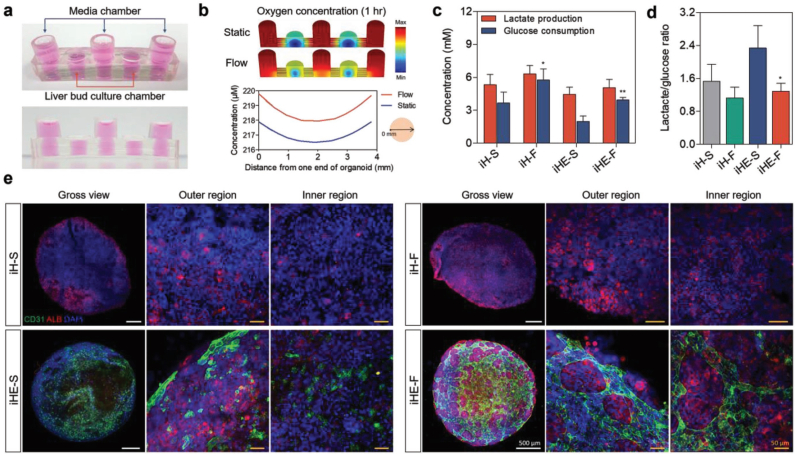
(a) Images illustrating the microfluidic device. (b) Oxygen concentration data simulation within the device under both static and fluidic flow conditions. (c) Intracellular production of glucose and lactate and (d) ratio of lactate to glucose in the 3D tissues within the microfluidic culture system. (e) Immunostaining for CD31 and albumin (ALB) in the 3D tissues. Induced hepatic cells were encapsulated in the liver ECM hydrogel with (iH) or without (iHE) human umbilical vein endothelial cells cultured under either static conditions (iH-S, iHE-S) or dynamic conditions (iH-F, iHE-F). Reproduced with permission from Ref. [123], Copyright © 2018 Wiley.

The dECM-based liver models serve as valuable tools for assessing the effects of diverse drugs. In an instance, Pati et al. observed that dECM preserves the phenotype of the HepG2 cell line while augmenting the expression of the metabolic marker carbamoyl phosphate synthetase I [122]. Under co-culture conditions with endothelial cells and stellate cells, both acetaminophen and ciprofloxacin demonstrated improved sensitivity, highlighting the influence of distinct cellular environments on drug-induced hepatotoxicity. In an instance, Cho and colleagues dynamically co-cultivated induced hepatic cells and endothelial cells with liver dECM hydrogel within a microfluidic culture system, thereby simulating the liver tissue environment and blood flow, consequently enhancing liver-specific functionality (Fig. 7) [123]. Hepatic cells in the presence of media flow in the 3D vascularized liver organoid exhibited heightened sensitivity and reduced variability in hepatotoxic response, rendering it conducive for toxicity assessments. In addition, it was observed that co-treatment with acetaminophen and ethanol enhanced the cytotoxic effects. Vosough et al. engineered controlled-size multicellular microtissues with acceptable basic hepatic characteristics and functions by co-culturing primary human hepatocytes, human mesenchymal stromal cells, and human umbilical vein endothelial cells with liver dECM microparticles for two weeks [124]. The primary human hepatocytes in liver microtissues incorporating liver ECM demonstrated superior hepatic gene expression and functional activity. Moreover, *in vitro* drug toxicity results indicated that the prepared microtissues incorporating liver ECM exhibited a higher dose-response sensitivity to diclofenac, 5-fluorouracil, acetaminophen, and tamoxifen compared to liver microtissues without the ECM. Rombouts and colleagues fabricated a decellularized human liver scaffold co-cultured with LX2 and HepG2 cells, enabling investigations into the anti-fibrotic of Sorafenib and the anti-cancer effects of Regorafenib [125]. The findings confirmed that Sorafenib's anti-fibrotic effect significantly mitigated the pro-fibrotic effect induced by TGF-β1 compared to 2D culture conditions. In addition, the 3D co-culture system based on liver ECM scaffolds exhibited higher resistance to Regorafenib treatment compared to 2D culture. The increased resistance might be related to the negative impact of the ECM microenvironment on cellular behavior, leading to a significant downregulation of Src homology region 2 domain-containing phosphatase 1 expression in human HCC tissues.

### 4.4 Others

Acute respiratory distress syndrome (ARDS) manifests as improved endothelial and epithelial permeability, culminating in lung parenchymal injury, often precipitated by bacterial or viral pneumonia. In a seminal study by Otero et al., a lung-on-chip device was deployed to cultivate single-layered alveolar epithelial cells on decellularized lung hydrogels enriched with pulmonary interstitial cells [126]. This setup faithfully mimicked breathing dynamics through cyclic stretching while emulating the respiratory milieu by inducing inflammation with bacterial toxins. Compared to traditional 2D models, this model showed that the anti-inflammatory drug dexamethasone showed no significant reduction in pro-inflammatory cytokines, which was more consistent with *in vivo* results. These findings suggested that the model more accurately reflected the clinical responses of patients.

Decellularized skeletal muscle holds significant promise in the realms of tissue engineering and regenerative medicine. Possessing tissue-specific morphological attributes, it serves as a reservoir of biochemical cues conducive to muscle cell regeneration [127]. Intramuscular injection stands as a prevalent route for drug delivery, wherein the drug's absorption within muscles and subsequent systemic circulation are critical factors. Consequently, the examination of drug-ECM interactions at the injection site within muscle tissue assumes paramount importance. A pioneering investigation led by Christman and colleagues fabricated decellularized skeletal muscle ECM material, with cobinamide chosen as a model drug to elucidate its interactions with the ECM [128]. Remarkably, the experimental findings demonstrated improved binding affinity of cobinamide with decellularized skeletal muscle ECM compared to collagen hydrogels and hyaluronic acid hydrogels, shedding light on its potential implications in drug delivery strategies.

**Fig. 8:**
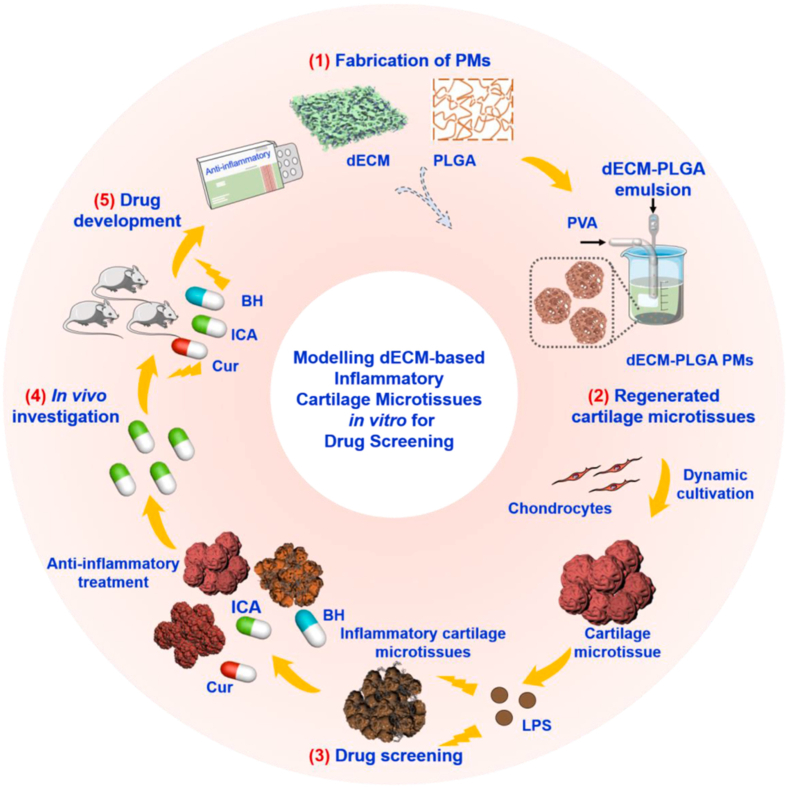
The construction of a multicellular aggregate cartilage inflammation model utilizing dECM-based PLGA porous microspheres. Reproduced with permission from Ref. [132], Copyright ©2023, Elsevier.

Bone is a dynamically intricate tissue that relies on highly orchestrated interactions among diverse bone cells, including osteoblasts, osteocytes, and osteoclasts. Moreover, the complexity of bone microenvironment poses significant challenges in developing appropriate bone chip platforms. In an attempt to address this, Kim and colleagues pioneered the development of a high-throughput biomimetic bone chip platform utilizing dECM derived from osteoblasts to emulate cellular-ECM interactions within the bone milieu [129]. The innovative design featured a circular chamber with dual compartments representing the structural properties of bone. Following treatment with osteoporosis drugs, β-catenin was predominantly localized in the cell nucleus, whereas in the untreated group, it was distributed in the cytoplasm. This device could be well-suited for the evaluation of osteoporosis treatments. Osteoarthritis (OA) is a common age-related degenerative joint disease primarily caused by chondrocyte apoptosis and an imbalance in extracellular matrix (ECM) synthesis and metabolism [130]. However, the *in vitro* osteoarthritis models were established using methods such as monolayer cell cultures, explant cultures, and multicellular co-cultures, with pro-inflammatory stimuli like inflammatory cytokines and mechanical loading inducing arthritis-like characteristics in chondrocytes [131]. Reliable osteoarthritis models are crucial for studying disease mechanisms and drug screening. In a recent breakthrough, Chen and group presented a novel method for fabricating large porous microspheres through the synergistic application of cartilage dECM and poly(lactic-co-glycolic acid) (PLGA) *via* microfluidic technology (Fig. 8) [132]. The subsequent dynamic cultivation of chondrocytes yielded a cartilage model, further augmented by the establishment of a cartilage inflammation model *via* lipopolysaccharide stimulation. Intriguingly, the therapeutic efficacy of three classic anti-inflammatory drugs—curcumin, icariin, and berberine hydrochloride—was meticulously examined, with icariin demonstrating superior anti-inflammatory properties, thereby holding promise for combating cartilage inflammation.

## 5 Future directions and conclusions

Previously, the U.S. Food and Drug Administration (FDA) and the European Medicines Agency (EMA) typically required new drugs to be evaluated in animal models before entering clinical trials. However, most candidate drugs were found to be unsafe or ineffective when transitioning from animal testing to human clinical trials. At the end of 2022, the FDA framed a policy change allowing new drugs to be approved without mandatory animal testing [133]. Although this may be implemented cautiously in the drug development process, it represents a trend where drug evaluation through computer modeling and *in vitro* models will become more prominent in the future. Indeed, regulatory authorities in an increasing number of countries and regions, including the United States, Europe, and India, are encouraging the use of computer simulations and *in vitro* models to improve regulatory efficiency and accelerate drug development [117]. *In vitro* models for clinical mimicry can be broadly categorized into two types: static 3D culture systems with complex structures and organ-on-chip models utilizing microfluidics. Currently, static 3D culture systems based on microengineered tissues and organ-on-chips play a crucial role in simulating disease states at the cellular and tissue levels and in assessing drug metabolism and toxicity. Meanwhile, organ-on-chip models have been developed to simulate almost all organ systems and associated diseases, providing new insights into the molecular biology and cellular basis of physiological and pathological processes [134]. Organ-on-chip models, which feature flowing fluids, are better able to simulate interstitial flow and other dynamic characteristics compared to static 3D models. These advancements provide important evidence for clinical simulation in new drug development and personalized medicine, representing the future direction of non-animal experimental methods.

ECM materials are derived from the native growth environment of cells and are a type of biomaterial with multiple beneficial functions and wide-ranging applications. In our recent study, we were the first to report the development of ECM-drug conjugates for targeted tumor therapy [135]. dECM offers a physiologically relevant milieu for constructing *in vitro* disease models, enabling more authentic drug response assessments. However, the intricate composition of ECM, comprising various components capable of influencing cellular states, presents challenges. The absence of specific components may introduce biases in drug screening outcomes.

On the one hand, different tissues may be suited to different decellularization techniques, making the process more efficient while minimizing alterations to ECM components, which requires experimental exploration. On the other hand, new decellularization techniques need to be developed. For example, supercritical carbon dioxide fluid technology is an efficient decellularization method that preserves cellular components better than surfactant-based elution methods. Moreover, vigilance is warranted regarding the potential impacts of incompletely cleared cellular remnants on cellular states within these models. Therefore, continuous advancements in decellularization techniques are necessary to effectively remove cellular components while maximizing the preservation of ECM components, representing the future pursuit of obtaining dECM materials. Efforts in drug screening models aim to closely mimic *in vivo* conditions, with ECM materials furnishing cells with surrounding components akin to their natural milieu while maintaining structural fidelity to *in vivo* cell contacts. For instance, perfusing decellularization solutions through the vasculature during organ decellularization endeavors to conserve original vascular architectures. Nevertheless, replicating *in vivo* dynamics such as blood and tissue fluid flow poses challenges *in vitro*. As a biomaterial, ECM offers advantages over other synthetic polymer materials due to its biological functions, while its physical properties, such as mechanical performance, are its shortcomings. For example, combining dECM materials with conductive synthetic materials can enhance the conductivity of cell scaffolds and regulate cellular behavior. Additionally, dECM materials related to bone are challenging to mimic the native hardness of bone, which significantly differs from the cellular growth environment in bone. Therefore, it is feasible to combine them with harder synthetic materials and inorganic calcium salts to enhance the mechanical strength of the culture scaffold. Currently, ECM gels are commonly used in the preparation of various organ-on-chip models. More precise utilization of different types of ECM materials is expected to achieve better outcomes. Additionally, the advent of organoids has significantly advanced drug screening efforts. Organoids are derived from stem cell differentiation into organ-specific cells. Incorporating ECM materials aims to provide organoids with matrix components similar to those in native tissues, and the involvement of various cell types in the model enhances its physiological relevance.

In the realm of personalized medicine, ECM-based scaffold materials hold significant promise. Typically, researchers face challenges in obtaining human-derived dECM materials and thus resort to decellularizing organ tissues from rodents or pigs. However, these cross-species ECM materials differ significantly in structure and the composition of supporting biomolecules from the human cellular environment. Furthermore, ECMs can vary between different individuals within the human population. Xie et al. confirmed differences in the content and structure of components such as pro-collagen I, fibronectin, and laminin in ECM derived from normal human breast tissue and two molecular subtypes of breast cancer. Subsequent cell culture studies revealed variations in gene expression, epithelial-mesenchymal transition (EMT) processes, and sensitivity to endocrine therapy among MCF-7 cells [97]. Leveraging dECM materials and matrix cells sourced from patients' excised tissues, personalized models can be tailored for drug screening in personalized therapy. For example, in tumor therapy, extracting tumor tissue from patients, isolating and extracting tumor cells and dECM materials, and subsequently using these fo*r in vitro* culture and drug screening for personalized treatment shows promising potential. However, this process faces two major challenges: first, it must be timed to match the progression of the patient's condition, and second, patient-derived dECM materials are often limited in quantity, leading to a lower margin for error.

In summary, with advancing insights into the multifaceted roles of ECM components, dECM materials are poised to emerge as pivotal scaffolds for cell models employed in drug screening. However, further refinement of decellularization techniques is imperative to better emulate the components and architectural nuances encountered by cells *in vivo* within drug evaluation models. Presently, ECM-based cell models exhibit substantial advantages over traditional synthetic materials in screening for anti-tumor drugs, as well as drugs pertaining to heart disease, liver disease, osteoarthritis, and various other conditions. Looking ahead, more precise acquisition of ECM materials and the development of *in vitro* models with greater cellular involvement, combined with advanced static or dynamic 3D culture techniques, are expected to drive advancements in drug screening and personalized treatment.

## CRediT authorship contribution statement

**Zhoujiang Chen:** Writing – original draft, Funding acquisition, Conceptualization. **Ji Wang:** Writing – original draft. **Ranjith Kumar Kankala:** Writing – review & editing, Validation. **Mingli Jiang:** Validation. **Lianlin Long:** Writing – original draft. **Wei Li:** Validation, Funding acquisition. **Liang Zou:** Validation, Funding acquisition. **Aizheng Chen:** Supervision, Project administration. **Ya Liu:** Supervision.

## Declaration of competing interest

The authors declare that they have no known competing financial interests or personal relationships that could have appeared to influence the work reported in this paper.

## Data Availability

Data will be made available on request.
